# Risk factors for cerebral vasospasm in patients with aneurysmal subarachnoid hemorrhage

**DOI:** 10.1515/med-2020-0169

**Published:** 2020-07-03

**Authors:** Valentina Opancina, Snezana Lukic, Slobodan Jankovic, Radisa Vojinovic, Milan Mijailovic

**Affiliations:** University of Kragujevac, Serbia, Faculty of Medical Sciences, Department of Radiology, Serbia; University of Kragujevac, Serbia, Faculty of Medical Sciences, Department of Pharmacology and Toxicology, Serbia

**Keywords:** cerebral vasospasm, endovascular coiling, aneurysm, international normalized ratio, white blood cells

## Abstract

**Introduction:**

Aneurysmal subarachnoid hemorrhage is a type of spontaneous hemorrhagic stroke, which is caused by a ruptured cerebral aneurysm. Cerebral vasospasm (CVS) is the most grievous complication of subarachnoid hemorrhage (SAH). The aim of this study was to examine the risk factors that influence the onset of CVS that develops after endovascular coil embolization of a ruptured aneurysm.

**Materials and methods:**

The study was designed as a cross-sectional study. The patients included in the study were 18 or more years of age, admitted within a period of 24 h of symptom onset, diagnosed and treated at a university medical center in Serbia during a 5-year period.

**Results:**

Our study showed that the maximum recorded international normalized ratio (INR) values in patients who were not receiving anticoagulant therapy and the maximum recorded white blood cells (WBCs) were strongly associated with cerebrovascular spasm, increasing its chances 4.4 and 8.4 times with an increase of each integer of the INR value and 1,000 WBCs, respectively.

**Conclusions:**

SAH after the rupture of cerebral aneurysms creates an endocranial inflammatory state whose intensity is probably directly related to the occurrence of vasospasm and its adverse consequences.

## 1 Introduction

Aneurysmal subarachnoid hemorrhage (ASAH) is a type of spontaneous hemorrhagic stroke, which is caused by a ruptured cerebral aneurysm in about 80% of patients with subarachnoid hemorrhage (SAH) [1,2]. Nearly one-third of ASAH survivors develop delayed cerebral ischemia, which is caused by the narrowing of cerebral blood vessels and decreased cerebral blood flow [3,4]. Segmental or diffuse narrowing of the lumen of the intracranial arteries is also known as cerebral vasospasm (CVS) [2,5]. CVS is the most grievous complication of SAH and can be described as a deferred and self-limiting condition; furthermore, its severity is associated with the volume, density, extended presence and site of subarachnoid blood [1,3,5]. CVS that is influenced by SAH is a perplexing issue which incorporates hypovolemia, damaged auto-regulatory function and also prolonged and reversible vasculitis [6,7]. The preeminent concept for CVS prevention is maintenance of regular blood volume as well as treatment with nimodipine [8]. Currently, the only confirmed treatment for CVS is euvolemic induced hypertension, due to the fact that endovascular procedures still carry specific risks [9].

To date, various factors have been known to be linked with an increased risk of CVS after SAH. The haptoglobin phenotype is one of these factors, since the affinity of the molecule for hemoglobin depends on the extent to which it will bind free hemoglobin in the subarachnoid space and thus prevent the initiation of a series of reactions that ultimately result in vasospasm formation [10]. Intraventricular hemorrhage within ASAH is also associated with a higher incidence of vasospasm, as well as long-term use of tobacco [11]. Also, left ventricular hypertrophy detected on electrocardiography is described as a risk factor for development of CVS (odds ratio [OR] = 3.48) [12]. The correlation between cerebrospinal fluid and CVS was observed, using volumetric analysis and SAH/CSF ratio (OR = 1.03) [13].

However, the amount of data published in this field to date has been relatively limited. Most of the studies explored CVS only in patients who developed neurological symptoms after the procedure [12,14,15]. Also, in a great number of other studies, the patient population was heterogeneous, including both those treated surgically and those treated by the endovascular approach [16,17].

Considering this, the aim of this study was to examine the risk factors that influence the onset of CVS that develops after endovascular coil embolization (EE) of a ruptured aneurysm which caused SAH.

## 2 Materials and methods

The study was designed as a cross-sectional study. It was approved by the Ethics Committee of the Clinical Center Kragujevac before any of the study procedures was initiated. The study was conducted according to the principles of Declaration of Helsinki about experimentation on human subjects and in compliance with national regulations. Informed consent was obtained from all individuals included in this study.

**Figure 1:**
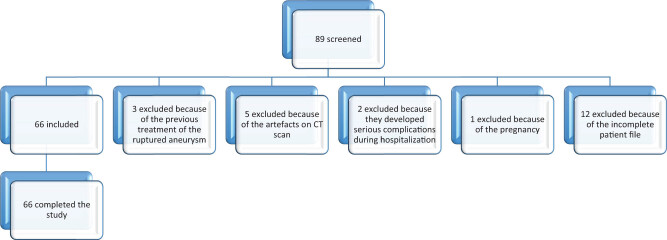
The study flowchart.

The patients included in the study satisfied the following criteria: 18 or more years of age, admitted within a period of 24 h of symptom onset, diagnosed and treated at Clinical Center Kragujevac, Serbia, during the observation period (from 01-01-2014 to 31-12-2018), and suffering from SAH caused by the rupture of an intracranial aneurysm for the first time; SAH confirmed by the CT scan on admission; rupture of an aneurysm confirmed by digital subtraction angiography (DSA); treatment of the aneurysm by EE; minimum two control CT scans after the coiling (the first CT scan was done 1 day after the endovascular procedure, the last CT was done on discharge and additional CT scans were done on demand from neurologists); and DSA after the embolization, between the 5th and 10th day. Each interventional radiology procedure was performed by the same team of two experienced neuroradiologists, and all the CT scans were evaluated by three neuroradiologists in our department. The exclusion criteria were the following: previous treatment of the ruptured aneurysm; artifacts in CT scans; patients who developed serious complications during hospitalization such as sepsis, hepatorenal dysfunction etc.; pregnant women; and incomplete patient file. Figure 1 presents the flowchart with the number of patients who were initially included and the number of excluded patients based on each of the exclusion criteria.

The diagnosis of ASAH was made if CT scan showed the presence of blood in the subarachnoid space, and DSA indicated rupture of an aneurysm. CVS was diagnosed by DSA [1,2] if the column of contrast in large cerebral arteries was decreased by more than 34% in diameter [13].

The following variables were extracted from the patient files: sociodemographic data: age, sex, residence, smoking habit, and caffeine use; symptoms and physical status prior to admission; scales: modified Rankin scale (MRS) on discharge [18], Hunt and Hesse scale (HHS) on admission [19], Fischer scale (FS) using the initial CT scan [20], and Glasgow Coma Scale (GCS) on admission; CT findings: hydrocephalus, brain edema, intraventricular hemorrhage, and intracerebral hemorrhage; liquid evacuation: lumbar puncture and/or ventriculoperitoneal shunt; mechanical ventilation after the endovascular procedure; aneurysm features: location, size, existence of a second unruptured aneurysm; procedure characteristics: duration of fluoroscopy and heparin dosage; duration of hospitalization, the time from admission to EE; duration of symptoms prior to hospital admission; and laboratory analysis (maximum recorded values and nadir values): blood count, coagulation tests, and biochemistry analysis.

### 2.1 Statistics

The study data were analyzed using SPSS version 23 software (SPSS Inc., Chicago, IL, USA) [21]. Descriptive statistics was used for primary data processing: continuous variables were described by mean and standard deviation (if normally distributed) or by median and interquartile range (if not normally distributed), while categorical variables were presented with rates and percentages. The Kolmogorov–Smirnov test was used to test the normality of the data distribution. Significance of differences in the study groups was tested by the Mann–Whitney test for continuous variables and by contingency tables for categorical variables. The impact of these variables on the main outcome (appearance of CVS) was investigated by univariate logistic regression at first, after which multivariable logistic regression was performed using the backward method. The model of logistic regression was built after several attempts, using the backward method; each attempt implied different combinations of independent and confounding variables. However, in order to avoid the co-linearity problem, only one value of the variables that were measured repeatedly (e.g. white cell count before, during and after the EE) was entered at a time (i.e. repeating measures of the same variable were never entered in the same model building attempt). Effects of the variables obtained in the final model were adjusted for their simultaneous presence. The quality of the multivariable logistic regression model was tested by Hosmer–Lemeshow, Cox–Snell and Nagelkerke tests.

## 3 Results

In total 66 patients were enrolled, and all of them completed the study, without drop-outs. The main characteristics of the study group are shown in Table 1, while for the sake of clarity the rest are listed in the supplementary file. The results of univariate logistic regression, for the outcome CVS, are demonstrated in Table 2. The univariate regression revealed that acute hydrocephalus after SAH, maximum recorded international normalized ratio (INR) and maximum recorded white cell count substantially increased the chances of CVS in post-embolization clinical course (ORs 5.000, 4.103 and 6.720, respectively). The influence of platelet count at nadir on CVS was statistically significant, but marginal (ORs between 1 and 1.01) (Table 2). Finally, the maximum recorded level of blood urea nitrogen was also significantly associated with CVS, with moderate influence (OR 1.285).

**Table 1:** Characteristics of the study population

Risk factors	Cerebrovascular spasm (*n* = 33)	No cerebrovascular spasm (*n* = 33)	*P* value
Age (mean ± SD, median [IQR])	55.45 ± 12.13, 56 [14]	52.55 ± 9.55, 54[17]	0.336
Age category (20–40/40–60/>60 years)	4/16/13 (12.1%/48.5%/39.4%)	5/21/7 (15.2%/63.6%/21.2%)	0.274
Gender (male/female, %/%)	8/25 (24.2%/75.8%)	10/23 (30.3%/69.7%)	0.580
Caffeine usage	23 (69.7%)	15 (45.5%)	0.046*
Impaired vision	1 (3%)	6 (18.2%)	0.046*
Mechanical ventilation	17 (51.5%)	8 (24.2%)	0.022*
Intraventricular hemorrhage	23 (69.7%)	13 (39.4%)	0.013*
Hydrocephalus	11 (33.3%)	3 (9.1%)	0.016*
Aneurysm size (<5 mm/5–10 mm/11–25 mm)	12/16/5 (36.4%/48.5%/15.2%)	6/20/7 (18.2%/60.6%/21.2%))	0.249
Aneurysm location (ACI/ACM/ACA/ACP/AB)	15/3/10/2/3 (45.5%/9.1%/30.3%/6.1%/9.1%)	10/7/13/2/1(30.3%/21.2%/39.4%/6.1%/3%)	0.407
Aneurysm height (mean ± SD, median [IQR])	6.718 ± 3.854, 5.52 [3.84]	8.099 ± 4.342, 7.08 [4.21]	0.099
Aneurysm width (mean ± SD, median [IQR])	5.496 ± 3.36, 4.45 [3.26]	6.63 ± 3.07, 6.29 [4.06]	0.045*
Aneurysm neck (mean ± SD, median [IQR])	3.43 ± 2.705, 2.70 [1.28]	2.902 ± 0.938, 2.78 [1.31]	0.812
GCS (mean ± SD, median [IQR])	11.15 ± 3.581, 13 [6]	11.09 ± 3.146, 12 [5]	0.640
HHS (mean ± SD, median [IQR])	2.82 ± 1.185, 3 [2]	3 ± 1.299, 3[2]	0.605
FS (mean ± SD, median [IQR])	3.45 ± 0.833, 4 [1]	3.03 ± 0.847, 3 [2]	0.029*
MRS (mean ± SD, median [IQR])	3.91 ± 1.569, 4 [4]	3.03 ± 1.447, 2 [2]	0.017*

*Statistically significant.

Abbreviations: ACI – a. carotis interna, ACM – a. cerebri media, ACA – a. cerebri anterior, ACP – a. cerebri posterior, AB – a. basilaris, GCS – Glasgow Coma Scale, HHS – Hunt and Hesse scale, FS – Fisher scale, IQR –  interquartile range, MRS – modified Rankin scale.

For the sake of clarity, the Mann–Whitney *U* test was done for continuous variables and the Chi-squared test for categorical variables.

**Table 2:** Univariate analysis of factors associated with CVS (majority of the factors without significant influence were omitted for the sake of clarity)

Risk factors	*P* value	Crude odds ratio	Confidence interval (95%)
Age	0.280	1.025	0.980–1.073
Gender	0.581	0.736	0.248–2.186
GCS score (≤8/>8)	0.148	0.411	0.123–1.373
Hydrocephalus (yes/no)	0.023	5.000*	1.245–20.076
Intraventricular hemorrhage (yes/no)	0.015	3.538*	1.277–9.805
Mechanical ventilation (yes/no)	0.025	3.320*	1.163–9.477
Maximum recorded CRP	0.164	0.444	0.142–1.394
Maximum recorded WBC	0.001	6.720*	2.080-21.708
PLT at nadir (×10^9^/L)	0.029	1.007*	1.001–1.012
Maximum recorded INR	0.008	4.103*	1.455–11.567
Proteins at nadir	0.999	0.000	0.000
Maximum recorded urea (mmol/L)	0.021	1.285*	1.039–1.590

* – statistically significant.

CRP – C reactive protein; WBC – white blood cell count; PLT – platelet count; INR – international normalized ratio.

The results of multivariable logistic regression are shown in Table 3. The estimates of the coefficient of determination according to the Cox and Snell and Nagelkerke were 0.398 and 0.531, respectively, while the Hosmer–Lemeshow test showed that the observed rate of CVS matched the expected rate of the same phenomenon (*χ* ^2^ = 2.503, *p* = 0.927). After adjustment, the following factors remained significantly associated with CVS: maximum recorded white cell count and INR. The strength of the association remained similar to that after univariate analysis, and the direction of the influence did not change (Table 3).

**Table 3:** Multivariate analysis of factors associated with CVS

Risk factors	*P* value	Adjusted odds ratio	Confidence interval (95%)
PLT at nadir (×10^9^/L)	0.099	1.007	0.999–1.014
Maximum recorded urea (mmol/L)	0.082	1.232	0.974–1.558
Maximum recorded INR	0.027	4.411*	1.181–16.478
Maximum recorded WBC	0.004	8.376*	2.009–34.921

* – statistically significant.

PLT – platelet count; INR – international normalized ratio; WBC – white blood cell count.

## 4 Discussion

Our study showed that the maximum recorded INR values in patients who were not receiving anticoagulant therapy and the maximum recorded WBC were strongly associated with cerebrovascular spasm, increasing its chances 4.4 and 8.4 times with an increase of each integer of the INR value and 1,000 white blood cells (WBCs), respectively.

Elevated INR values in patients not receiving anticoagulant therapy were recently linked to an increased risk of intracerebral aneurysm rupture, although the possible mechanism of action was not proposed [22]. A similar effect was not observed in patients on anticoagulant therapy, suggesting the protective role of oral anticoagulants independent of their main pharmacological effect (vitamin K antagonism) [23]. Although systemic inflammation creates a hypercoagulable state through activation of coagulation, decrease of endogenous anticoagulants in blood, and inhibition of fibrinolysis [24], INR is usually elevated due to the consumption of certain proteins involved in the coagulation cascade [25]. Elevated INR observed in our study may reflect a similar influence of systemic inflammation (C reactive protein was elevated in almost 72% of our patients) on the coagulation cascade. Inflammation was already associated with ASAH (elevated interleukin levels), and it was shown that its highest intensity was localized to the brain tissue, while changes in the blood are not that pronounced [26]. Increased levels of interleukin 6 were also found in patients with ASAH who developed CVS [27]. Therefore, elevated INR values are probably associated with intense inflammation of the brain tissue after the rupture of an aneurysm that will cause the release of a multitude of autacoids. At least some of the released autacoids may activate the receptors on smooth muscle cells and produce intense vasospasm, such as cysteinyl leukotrienes [28].

The elevated WBC count after the percutaneous intervention is also reflection of the inflammatory process in the brain tissue, and its association with CVS after SAH was not surprising. It was recently understood that a lot of vascular and neural changes after SAH were caused by inflammation accompanied by the activation of immune cells in brain parenchyma and blood [29]. There are many autacoids, hormones and neurotransmitters that may cause vasospasm, and some of them are released from platelets, such as serotonin, while the others could originate from other blood cells destroyed after SAH [30,31]; injured neurons also react with increased firing, so local concentrations of vasoactive catecholamines, endothelins or other substances may mount, causing intense and prolonged vasoconstriction.

Although mechanical ventilation, hydrocephalus and intraventricular hemorrhage were more frequent among patients in our study who developed cerebrovascular spasm, after adjustment for confounding factors their influence on spasm did not reach statistical significance. Intraventricular hemorrhage was previously linked with inflammation of the brain tissue in several animal studies, causing an upregulation of pro-inflammatory cytokines, attracting WBCs and activating microglia [32]. On the other hand, hydrocephalus could be a consequence of intraventricular hemorrhage accompanying inflammation and edema. However, intraventricular hemorrhage probably was not the sole cause of neuroinflammation and release of mediators that produced vasoconstriction in our patients, which, together with a relatively small number of patients in the study, could explain why it was not among the significant risk factors after adjustment and multivariate analysis.

Although caffeine usage in our study was not significantly associated with CVS, some authors did find an association between them [33]. A probable explanation for caffeine-induced CVS is a pro-thrombotic state with decreased ability of blood vessels to dilate, since caffeine from caffeine-rich beverages or coffee increases platelet aggregation and alters endothelial function reducing the ability of the endothelium to mediate vascular relaxation. However, further research is necessary to confirm this hypothesis.

The size of cerebral artery aneurysms was previously connected with the rate of cerebral artery vasospasm after SAH [14], but this is an indirect effect, since CVS is more frequent when the extent of subarachnoid bleeding is large. However, there are no studies finding an association between the size of aneurysms and rate of CVS after endovascular embolization. Our study also did not find such an association, but this could be a consequence of insufficient statistical power, implying the necessity for continuous research on this issue.

The limitation of our study was a relatively small sample size, which allowed simultaneous testing of only up to ten study variables in multivariate logistic regression, increasing the chances of statistical type 2 error. Our study was unicentric, which is an independent risk factor for introducing local practice bias in the interpretation of the results.

In conclusion, SAH after rupture of cerebral aneurysms creates an endocranial inflammatory state whose intensity is probably directly related to the occurrence of vasospasm and its adverse consequences. Whether pharmacological interference for neuroinflammation could be a useful strategy for the prevention of vasospasm remains to be decided by future studies of sufficient size.
